# Biomechanical Behavior of Bioactive Material in Dental Implant: A Three-Dimensional Finite Element Analysis

**DOI:** 10.1155/2020/2363298

**Published:** 2020-05-07

**Authors:** Vathsala Patil, Nithesh Naik, Srikanth Gadicherla, Komal Smriti, Adithya Raju, Udit Rathee

**Affiliations:** ^1^Department of Oral Medicine and Radiology, Manipal College of Dental Sciences Manipal, Manipal Academy of Higher Education, Manipal, Karnataka, India; ^2^Department of Mechanical and Manufacturing Engineering, Manipal Institute of Technology, Manipal Academy of Higher Education, Manipal, Karnataka, India; ^3^Department of Oral and Maxillofacial Surgery, Manipal College of Dental Sciences Manipal, Manipal Academy of Higher Education, Manipal, Karnataka, India; ^4^Medical Engineering, KTH Royal Institute of Technology, University in Stockholm, Brinellvägen 8, 114 28 Stockholm, Sweden

## Abstract

Dental implants are widely accepted for the rehabilitation of missing teeth due to their aesthetic compliance, functional ability, and great survival rate. The various components in implant design like thread design, thread angle, pitch, and material used for manufacturing play a critical role in its success. Understanding these influencing factors and implementing them properly in implant design can reduce cases of potential implant failure. Recently, finite element analysis (FEA) is being widely used in the field of health sciences to solve problems in designing medical devices. It provides valid and accurate assessment in the clinical and in vitro analysis. Hence, this study was conducted to evaluate the impact of thread design of the implant and 3 different bioactive materials, titanium alloy, graphene, and reduced graphene oxide (rGO) on stress, strain, and deformation in the implant system using FEA. In this study, the FEA model of the bones and the tissues are modeled as homogeneous, isotropic, and linearly elastic material with a titanium implant system with an assumption of it 100% osseointegrated into the bone. The titanium was functionalized with graphene and graphene oxide. A modeling software tool Catia® and Ansys Workbench® is used to perform the analysis and evaluate the von Mises stress distribution, strain, and deformation at the implant and implant-cortical bone interface. The results showed that the titanium implant with a surface coating of graphene oxide exhibited better mechanical behavior than graphene, with mean von Mises stress of 39.64 MPa in pitch 1, 23.65 MPa in pitch 2, and 37.23 MPa in pitch 3. It also revealed that functionalizing the titanium implant will help in reducing the stress at the implant system. Overall, the study emphasizes the use of FEA analysis methods in solving various biomechanical issues about medical and dental devices, which can further open up for invivo study and their practical uses.

## 1. Introduction

Dental implants are widely accepted by patients for the rehabilitation of partially edentulous arches due to its aesthetic compliance, functional ability, and great survival rate [1]. The appropriate selection of implant design is critical, as it plays an important role to preserve the osseointegration [2]. The success of the dental implant and osseointegration of bone-implant are influenced by several elements including material biocompatibility, implant design, surface treatment, surgical technique, bone quality, and loading conditions [3, 4]. In an attempt to study the biomechanical factors, researchers have targeted the implant macrodesign that affects the long-term success of the implant.

The thread in the implant acts as a retentive element, increasing the contact surface area to provide greater bone-implant interaction, thus providing better stress distribution [5–8]. The thread design, the pitch of the implant, and the material of implant help to determine the longevity and primary stability of the implant under immediate loading conditions [9, 10]. The high mechanical strength, corrosion resistance, fracture toughness, and excellent biocompatibility property make titanium alloys the most preferred and established biomedical material for implant fabrication [11, 12]. However, it is observed that the formation of an oxidized layer on its surface obstructs the interface of the implant and contacting bone tissues leading to inadequate integration with the adjoining bone. The formation of this biofilm also leads to increased susceptibility to infection, therefore leading to implant failure [13].

Several kinds of research have explored implants coated with hydroxyapatite (HA) and have recommended that, for anterior and posterior maxilla, HA-coated screw implants should be used where the depth of the bone reaches 10 mm and where the cortical layer is thinner. When the cortical layer of the posterior maxilla is thin and of low density, it is recommended to have an HA-coated cylindrical implant. However, the use of HA-coated implants is a concern, as the clinician should consider bacterial susceptibility of HA-coated implants in comparison with titanium implants. Besides, failure may also be observed due to coating-substrate fracture [14, 15].

Further studies led to exploring newer composite materials in dental applications termed as bioactive materials that provide varied properties. The surface coating or functionalizing of the implant with different suitable bioactive materials like graphene [16] and reduced graphene oxide (rGO) has displayed effective improvement in implant biocompatibility with jawbones. The bioactive materials proven to have antibacterial property promote regeneration of bone tissue and increase bond strength at the interface of the implant with the bone [17].

Recently, many studies have also focused on modification of implant design by making alterations in some of the geometric patterns such as thread pitch, helix angle, depth, and width. Amid the different implant design variables taken under consideration, the pitch is contributing significantly to stability by imparting an effect on the surface area [14–20].

Mehrali et al. [21] studied the stress distribution and stability of bioactive material implants. The functionalizing of the screw reduced the stress at implant and jaw bone interface significantly. Rahbar et al. [22] study showed a reduction in the stress with the increase of critical crack length. Sadollah et al. [23] also performed a finite element analysis of functionalized implants to reduce stress concentration. Several studies have also analyzed the properties of the dental implants made of functionally graded biomaterials (FGBMs), developed to satisfy the heterogeneity of the tissue and proved to have good osseointegration. FGBM decreases the stress differential at the implant-interfaces effectively where maximum stress occurs but has a relatively low influence on all the system's natural frequencies [24–27]. Thus, understanding the effects of influencing factors and implementing them properly in the discipline of dental implants can help in reducing the potential implant failure.

To assess the aforementioned parameters, the numerical finite element analysis (FEA) provides valid and accurate assessment in the clinical and in vitro analysis. The FEA tool can be effectively used to evaluate the biomechanical behavior of the bone-implant interface and identify the regions of higher stress concentration. FEA models have provided an accurate three-dimensional insight into the phenomena occurring at the bone-implant interface over 2D axial-symmetric models [28].

In the present study, the influence of the thread design and bioactive materials, titanium alloy, grapheme, and reduced graphene oxide (rGO) considered on stress, strain, and deformation in the implant system is evaluated using three-dimensional (3D) FEA. Also, statistical analysis was performed to identify the parameters that significantly contribute to induce stress and strain concentrations.

## 2. Materials and Methods

### 2.1. Experimental Design and FE Model Generation

The present study involves assumptions made for material properties, model design, and FE model generation. The assumptions made directly influence the accuracy of the values derived from the FE analysis [28]. The bones and the tissues are modeled as homogeneous, isotropic, and linearly elastic, even though they are anisotropic materials [29, 30]. The study considers the osseointegration of bone-implant surface to be 100%, and the mandible section modeled is composed of cancellous bone surrounded by a cortical bone layer of 2 mm [30]. The mechanical properties of components of dental implant structure were taken from the literature [28].

Graphene is a two-dimensional form of carbon with distinctive mechanical, chemical, optical, and electrical properties. It has asymmetric nanostructure, which contributes to its rigidity and roughness. It also enhances the osteogenic differentiation of intraosseous human mesenchymal stem cells [31]. Reduced graphene oxide (rGO) is manufactured by the reduction of graphene by chemical or biological methods.

The reduction of GO can be carried out by using different reducing agents, which will lead to the production of chemical compositions of rGO in various C : O ratios [32]. Recently, the synthesis of nanoparticle rGO by using biological materials has received much attention due to their fewer energy requirements for manufacture, eco-friendly nature, durability, low cost, stability, and obtainability of the required solutions at high densities compared with chemical synthesis [33]. Graphene and its derivatives are functional materials that can be deposited onto different substrates and have enhanced physicochemical and mechanical properties, and hence confer increased bioactivity and new capabilities in existing biomaterials [34–36].

Table 1 shows the mechanical properties of the components and bioactive materials considered for the design of the dental implant.

### 2.2. Numerical Analysis

The ideal osseointegration is an assumption of perfect bond with no relative motion along with the interfaces of implant, bones, and abutments of the dental implant system. An ideal implant preload of 0.2 Nm in an anticlockwise direction and axial load (100 N, 150 N, 200 N, and 250 N) is applied to the occlusal surface of the crown [37]. The models were fully constrained for all degrees of freedom, i.e., all directions at the bone surfaces. In the present study, 3-dimensional FE models as per the grouping as shown in Table 2 are generated. The modeling software tool Catia® and Ansys Workbench® is used to perform analysis.

A convergence study is performed, by meshing the designed model with tetrahedral elements, with each node having three degrees of freedom of the implant system with coarse, medium, and fine mesh with the variable number of elements. The convergence of results was examined with a tolerance of 1% for von Mises stress at the cortical bone in the modeled implant system with a variable number of elements under the vertical loading condition. The convergence was observed for a refined mesh of the crown restoration, abutment, screw implant, and cancellous bone with an element size of 0.5 mm, whereas the cortical bone was the medium-mesh size.

### 2.3. Statistical Analysis

Using von Mises stress criterion, the qualitative 3D FEA was performed for the implant, and the data obtained for stress, strain, and deformation in the implant system as a whole and at the implant-bone interface were analyzed qualitatively. All the combinations of pitch group and biocompatible materials group sets were considered for analysis. The data obtained from FE analysis were analyzed for the groups and compared using Kruskal–Wallis statistical methods. The analysis is performed to calculate the significance of parameters and their contribution towards the stress concentration in the dental implant system [38, 39]. The materials 1, 2, and 3 considered for the analysis are titanium, graphene, and rGO, respectively, with pitches 1, 2, and 3 of standard single thread pitch of 1.0 mm, 1.4 mm, and 2.2 mm, respectively.

## 3. Results

**Figure 2:**
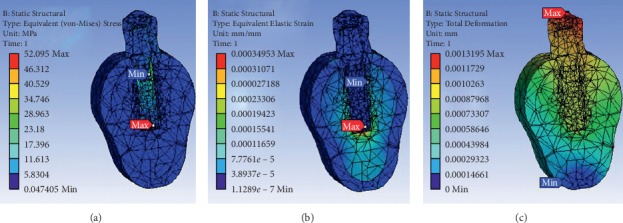
FE analysis in a functionalized implant with graphene as a biomaterial.

**Figure 3:**
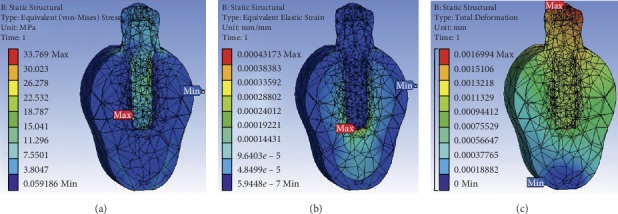
FE analysis in a functionalized implant with reduced graphene oxide as a biomaterial.

The prosthesis material, implant material, bioactive material coating, and thread designs greatly influence the stress distribution, strain concentration, and deformation in the bone around implants. The results obtained by FE analysis to evaluate the related properties are shown in Tables 3–5, for the implant system. Figures 2 and 3 show the von Mises stress, von Mises strain, and deformation in the dental implant for the FE analysis performed for the functionalized implant with graphene and reduced graphene oxide as biomaterial, respectively.

The Kruskal–Wallis test was applied to find the statistical difference among the groups. Tables 3–5 state the von Mises stress, strain, and deformation at the implant system for titanium, reduced graphene oxide, and graphene in three different pitch designs. Tables 6–8 show the Kruskal–Wallis analysis for different combinations of implant design and their stress distribution, strain, and deformation. Statistically, a significant difference was seen for stress (*p*=0.037), whereas there was no significant difference seen with deformation and strain.

Mean stress was found to be higher with pitch 2 (8.57 ± 2.87) when evaluated in titanium. The Kruskal–Wallis test applied to find the statistical difference among the groups (pitches 1, 2, and 3) at the cortical bone system for stress is presented in Table 7. It shows a statistically significant difference in stress (*p*=0.006).

Table 8 shows the results of the Kruskal–Wallis test applied to find the statistical difference among the groups (materials 1, 2, and 3) with different pitches (1, 2, and 3) at the implant system for stress. No significant difference was noted. The Kruskal–Wallis test applied to find the statistical difference among the groups (materials 1, 2, and 3) with different pitches (1, 2, and 3) at the cortical bone system for stress is depicted in Table 9. A statistically significant difference was seen for pitch 1 but no significant difference was seen in pitch types 2 and 3.

## 4. Discussion

Analyzing and attaining a logical solution to problems associated with the complex geometrical structure like bone and implant surface is challenging. In situations like this, computational tools such as finite element analysis (FEA) can be applied. FEA was first developed in the early 1960s to solve structural problems in the field of aerospace. It has been adapted from the engineering field and is widely applied in the field of dentistry as a prediction tool, mostly in understanding biomechanics of dental implants. Weinstein et al. employed this in implant dentistry for the first time. In the previous studies, FEA has been applied for the analysis of stress distribution patterns in a single tooth implant, to study various stages of bone and implant interface development [40]. FEA models have been developed for Osseointegrated cylindrical implants and cantilevered prostheses on dental implants [41, 42].

von Mises stress criterion calculates the combination of the stress that leads to failure at a particular point. The von Mises stress depicts the combined effect of three principal stresses into equivalent stress, which is compared with the yield stress of the material. This yield stress is the known property of the material and is generally considered to evaluate the failure stress for a given material. Thus, researchers consider von Mises stress as a criterion to evaluate the failure stress in dental studies [43]. In the present study, von Mises stress is considered to evaluate and observe the region of higher stress concentration in the dental implant system, as it estimates quantitatively the stress-induced at a point as a nonuniaxial stress rate for the bioactive material and thread design combinations considered.

In an implant system, the occlusal loads are directly transmitted to surrounding bone unlike the natural teeth and its supporting periodontal ligament fibers. This is the cause of failure in implants like fracture of the implant, loosening of components, and resorption of bone. Studies have attempted to reduce the implant failures by devising methods for stress distribution by introducing the contact area of bone and implant interface, increasing the diameter of the implant, length of the implant, and altering its design. Thread configuration is an important objective in biomechanical optimization. Threads are used to maximize initial contact, improve initial stability, enlarge implant surface area, and favor dissipation of interfacial stress [40, 44]. Out of various thread parameters like thread face angle, thickness, and pitch, the pitch has shown more clinical significance. Microthread designs are usually perceived to reduce the cortical bone loss and enhance osseointegration in supportive of mechanical stress theory which states that a mild overload at the thread crest activates osteoblasts to initiate bone formation [45–47].

The current study revealed the pitch of 1.5 mm to have low von Mises stress in the range of 10.5 MPa to 30.7 MPa at the implant and cortical bone interface when tested in three different materials. The results were comparable with the previous studies by Kong L et al. who also suggested a reduction in stress when the pitch was more than 0.8 mm. However, in our study, as the pitch increased to 2.2 (pitch 3), the von Mises stress was also greater than that before. Hence, an optimum pitch in the range of 1.5–2.2 (not more than 2.2) is a good combination to manage stress distribution and prevent implant failures [48].

Titanium is a commonly used implant material in the field of orthopedic and dental. It has good biocompatibility and high corrosion resistance. However, it has poor shear strength, which might lead to poor wear and tear. Titanium has been witnessed to have altered osteoinductivity and osteoconductivity impeding tissue regeneration [49, 50].

An in vivo study on various treatment methods has been advocated to increase the osteoblast responses. Carbon nanomaterials, carbon nanotubes, and graphene have been experimentally tested in implants. Graphene and rGO as coating materials have increased the success rate of dental implants due to its properties like high surface area and biocompatibility. Modified graphene sheets on titanium were used by Rojas and Leiva in 2007 for orthopedic implants [51]. Experiments with graphene modification on a dental scaffold of nickel-titanium have exhibited enhanced osseointegration and blood compatibility. Studies have also shown graphene oxide to have antibacterial properties. The adhesion of *S. aureus* was reduced when graphene oxide was reduced on titanium [52]. In vivo work on calvarial defects on the mouse using graphene also showed increased bone formation with lamellar formation [52].

RGO coating on the Ti surface could be easily performed not only on a 2D flat surface but also on a 3D structure, especially a screw-shaped implant. The functionalized and drug-loaded implant materials help to improve the cell reaction across the implant surface to reduce the time of osseointegration [53, 54].

The present work discusses the potential applications of graphene and rGO with titanium dental implants and stress, strain concentrations, and deformation at the bone and implant interface. The titanium dental implant with a surface coating of graphene oxide exhibited better mechanical behavior than graphene, with mean von Mises stress of 39.64 MPa in pitch 1, 23.65 MPa in pitch 2, and 37.23 MPa in pitch 3. This result shows that functionalizing the titanium implant will help in reducing the stress at the implant system. However, when 3 pitch designs were compared for deformation and strain, no significant changes were noted.

## 5. Limitations of the Study

The FE analysis has certain limitations that include varied mechanical properties of the bones for every individual case and nonlinear behavior of the tissues. Hence, more clinical trials are required to validate the findings of the present study.

In this study, stress analysis was done on the implant system as a whole and at cortical bone and implant interface. Further studies at the implant and cancellous bone also can provide a better understanding of stress distribution in various implant models.

## 6. Conclusions

The present study focuses on the investigation of the effect of functionalized titanium implant with graphene and reduced graphene oxide on stress distribution. The conclusions are summarized below:

1. The potential applications of graphene and rGO are discussed in dental implant applications
2. The functionalized implant has a positive effect on the reduction of stress in the implant
3. Through functionalization of the implant using biomaterials, the implant gradually converts from titanium with higher Young's modulus to a varied material at the interface
4. Although in vitro studies show positive results, in vivo research supporting the studies about tissue generation and osseointegration is essential to prove its potential application in dental implants
5. The future researchers on dental implants and dental tissue engineering will have graphene and rGO biomaterials in greater focus

## Figures and Tables

**Figure 1:**
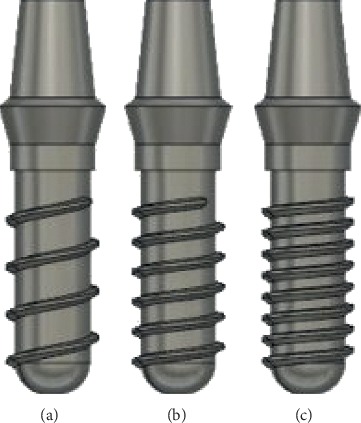
Configurations of implants with an abutment. (a) Single threaded 2.2 mm pitch. (b) Single threaded 1.4 mm pitch. (c) Single threaded 1 mm pitch.

**Table 1:** Mechanical properties of components and bioactive materials of the dental implant [28].

Component	Young's modulus (GPa)	Poisson ratio	Source
Cancellous bone	1.37	0.23	[31]
Cortical bone	13.7	0.3	[31]
Crown (porcelain)	68	0.35	[31]
Titanium	102	0.35	[31]
Graphene	1000	0.35	[32]
Reduced graphene oxide (rGO)	250	0.3	[33]

**Table 2:** Experimental grouping for the FE Analysis.

Group number	Group name	Description	Component
1	Pitch group	Standard pitch	Single thread
			1.0 mm, 1.4 mm, 2.2 mm (Figure 1)

2	Material group	Biocompatible material	Titanium
			Graphene
			Reduced graphene oxide (rGO)

**Table 3:** von Mises stress in three different pitches and three materials at the implant system and cortical bone and implant interface.

Pitch types	Material	Stress at the implant system				Stress at the cortical bone and implant interface			
		Load applied				Load applied			
		100 N	150 N	200 N	250 N	100 N	150 N	200 N	250 N
Pitch 1 (1 mm)	Titanium	17.2	25.8	34.4	43.1	4.734	7.128	9.403	11.79
	Reduced graphene oxide (RGo)	22.75	34.13	45.51	56.1	0.823	0.924	1.67	1.473
	Graphene	28.2	42.3	56.4	70.6	1.854	2.108	1.93	1.89

Pitch 2 (1.4 mm)	Titanium	10.5	15.8	21.2	26.4	5.16	7.71	10.305	12.77
	Reduced graphene oxide (RGo)	13.5	20.2	27.1	33.77	4.89	7.39	9.75	12.29
	Graphene	20.7	31.1	41.5	51.91	4.66	7.00	9.28	11.64

Pitch 3 (2.2 mm)	Titanium	16.65	24.9	33.3	41.6	4.3	6.42	8.52	10.761
	Reduced graphene oxide (RGo)	21.87	31.7	42.3	52.9	4.075	6.11	8.21	10.225
	Graphene	26.0	39.0	52.0	65.1	3.89	5.85	7.85	9.82

**Table 4:** Strain in the implant system in three different pitch designs of implant and three materials.

Pitch types	Material	Strain			
		Load applied			
		100 N	150 N	200 N	250 N
Pitch 1 (1 mm)	Titanium	0.000160	0.000240	0.000321	0.000401
	Reduced graphene oxide (RGo)	0.000162	0.000247	0.000330	0.000413
	Graphene	0.000170	0.000255	0.000340	0.000425

Pitch 2 (1.4 mm)	Titanium	0.000162	0.000246	0.000328	0.000411
	Reduced graphene oxide (RGo)	0.000172	0.000259	0.000345	0.000431
	Graphene	0.000178	0.000267	0.000356	0.000445

Pitch 3 (2.2 mm)	Titanium	0.000154	0.000231	0.000308	0.000385
	Reduced graphene oxide (RGo)	0.000161	0.000242	0.000323	0.000404
	Graphene	0.000174	0.000262	0.000349	0.000436

**Table 5:** Deformation in the implant system in three different pitch designs of implant and three materials.

Pitch types	Material	Load applied			
		100 N	150 N	200 N	250 N
Pitch 1 (1 mm)	Titanium	0.007	0.0010	0.0014	0.0017
	Reduced graphene oxide (RGo)	0.0006	0.0010	0.0013	0.0017
	Graphene	0.0006	0.0009	0.0013	0.0016

Pitch 2 (1.4 mm)	Titanium	0.00069	0.001	0.0013	0.0017
	Reduced graphene oxide (RGo)	0.00067	0.0010	0.0013	0.0017
	Graphene	0.00066	0.0009	0.0013	0.0016

Pitch 3 (2.2 mm)	Titanium	0.0007	0.0010	0.0013	0.0017
	Reduced graphene oxide (RGo)	0.0006	0.0009	0.0013	0.0016
	Graphene	0.0006	0.0010	0.0013	0.0016

**Table 6:** Comparison of stress, deformation, and strain in 3 pitch designs using Kruskal–Wallis at the implant system.

At implant system	Pitch	*N*	Minimum	Maximum	Mean	SD	Kruskal–Wallis	*p* value
Stress	1	12	17.20	70.61	39.73	15.77	6.608	0.037^*∗*^
	2	12	10.56	51.91	26.16	11.98		
	3	12	16.65	65.11	37.31	14.37		

Deformation	1	12	0.0006	0.0017	0.001150	0.00040	0.041	0.98
	2	12	0.0007	0.0017	0.001152	0.00038		
	3	12	0.0006	0.0017	0.001133	0.00039		

Strain	1	12	0.000160	0.000425	0.00028	0.000097	0.47	0.78
	2	12	0.000162	0.000445	0.00030	0.00010		
	3	12	0.000154	0.000436	0.00028	0.000097		

^*∗*^Significant.

**Table 7:** Comparison of stress in 3 pitch designs using the Kruskal–Wallis test at the cortical bone-implant interface.

Cortical bone	Pitch	*N*	Minimum	Maximum	Mean	SD	Kruskal–Wallis	*p* value
Stress	1	12	0.82	11.79	3.81	3.66	10.39	0.006^*∗*^
	2	12	4.66	12.77	8.57	2.87		
	3	12	3.89	10.76	7.16	2.42		

^*∗*^Significant.

**Table 8:** Kruskal–Wallis analysis of stress showing the comparison between pitch designs and materials at the implant system.

At implant system	Material	*N*	Minimum	Maximum	Mean	SD	Kruskal–Wallis	*p* value
Pitch 1	1	4	17.20	43.10	30.13	11.14	2.46	0.29
	2	4	28.24	70.61	49.42	18.23		
	3	4	22.75	56.19	39.64	14.42		

Pitch 2	1	4	10.56	26.40	18.50	6.83	3.96	0.13
	2	4	20.77	51.91	36.34	13.40		
	3	4	13.50	33.77	23.65	8.73		

Pitch 3	1	4	16.65	41.62	29.13	10.74	2.19	0.33
	2	4	26.04	65.11	45.57	16.81		

**Table 9:** Kruskal–Wallis analysis showing the comparison between pitch designs and materials at cortical bone and implant interface.

At cortical bone	Material	*N*	Minimum	Maximum	Mean	SD	Kruskal–Wallis	*p* value
Pitch 1	1	4	4.73	11.79	8.263	3.02	9.84	0.007^*∗*^
	2	4	1.85	2.10	1.945	0.11		
	3	4	0.82	1.67	1.222	0.41		

Pitch 2	1	4	5.16	12.77	8.986	3.28	0.61	0.73
	2	4	4.66	11.64	8.145	2.99		
	3	4	4.89	12.29	8.580	3.17		

Pitch 3	1	4	4.30	10.76	7.500	2.77	0.61	0.73
	2	4	3.89	9.82	6.852	2.55		
	3	4	4.07	10.22	7.155	2.65		

^*∗*^Significant.

## Data Availability

The data used in this are available from the corresponding author upon request.
